# Dataset of a study investigating autologous blood patch pleurodesis in postoperative prolonged air leaks after lung resection

**DOI:** 10.1016/j.dib.2020.105789

**Published:** 2020-06-04

**Authors:** Till Ploenes, Ioanis Kyritsis, Sandra Kampe, Khaled Mardanzai, Linda Langehegermann, Alexis Slama, Balazs Hegedüs, Clemens Aigner

**Affiliations:** a Department of Thoracic Surgery and Thoracic Endoscopy, Ruhrlandklinik, West German Lung Center, University Hospital Essen, University Duisburg-Essen, Essen, Germany; b Department of Anesthesiology, Ruhrlandklinik, West German Lung Centre, University of Duisburg-Essen, Essen, Germany and Department of Anesthesiology and Intensive Care Medicine Germany

**Keywords:** Thoracic surgery, Pain medication, Prolonged air leak, Blood patch pleurodesis, VAS

## Abstract

Prolonged air leak (PAL) after pulmonary resection is one if the most common complications in thoracic surgery. The dataset was obtained from a prospective randomized study investigating autologous blood patch pleurodesis in PAL. Patients were randomized to either receiving 100 ml autologous blood injected at postoperative days five and six (group A) or to watchful waiting (group B). The primary and secondary endpoints focused on differences in the duration of PAL in each group and possible complications. The results were reported in The Journal of Surgical Research. In this Data in Brief article, we provide additional data concerning pain medication and pain score during the first ten postoperative days. This should provide additional insights into the trial.

Specifications table

**Table:** 

Subject	Surgery
Specific subject area	Thoracic Surgery
Type of data	Table and Figures
How data were acquired	Medical records of all patients included in the prospective, randomized trial. The study was approved by the local ethics committee (16–6873-BO) and registered in the german clinical trial registry (DRKS00010211).
Data format	Raw baseline data
Parameters for data collection	Baseline parameters of pain medication and pain score at time of first day after operation until the 10th day after operation.
Description of data collection	Data were prospectively collected.
Data source location	City/Town/Region: Ruhrlandklinik, University Hospital Essen, University Duisburg-Essen, Essen, Germany Country: Germany
Data accessibility	With the article
Related research article	A prospective, randomized study investigating autologous blood patch pleurodesis in postoperative prolonged air leaks after pulmonary resection. Till Ploenes MD, Ioanis Kyritsis MD, Khaled Mardanzai MD, David Muhmann, Linda Langehegermann, Alexis Slama MD, Balazs Hegedüs PhD, Clemens Aigner MD [1]

## Value of the data

• Our prospectively collected data may be useful for clinicians and researchers working in the fields of thoracic surgery or pneumology. The data may also be useful for pain research.
• The data provide an insight in details of pain medication after lung surgery.
• The dataset provides a valuable starting point for further prospective studies in the field of thoracic surgery.

## Data description

This dataset contains rare data of a surgical cohort with PAL treated with autologous blood patch pleurodesis or watch and wait. We evaluated postoperative patient data on a daily basis, starting with the first postoperative day and up to 10 days postoperatively. This corresponds to day 5 after randomization. The dataset includes baseline data like gender, age, pain medication on discharge and pre-existing pain medication as well (Table 1).

**Tab. 1:** Patient characteristics.

	Group A (*n* = 10)	Group B (*n* = 14)
Female/Male	2/8	6/8
Median age	65.6 years (range 50.4–78)	66.6 years (range 56.2–79.1)
Pain Medication on discharge	Opioid* plus NSAR 6	Opioid* plus NSAR 7
	NSAR only 4	NSAR only 4
	None 0	None 3
Pre-existing pain medication	Opioid 1	None 14
	None 9	
		
		

⁎ ***low potential opioid***.

**Fig. 1:**
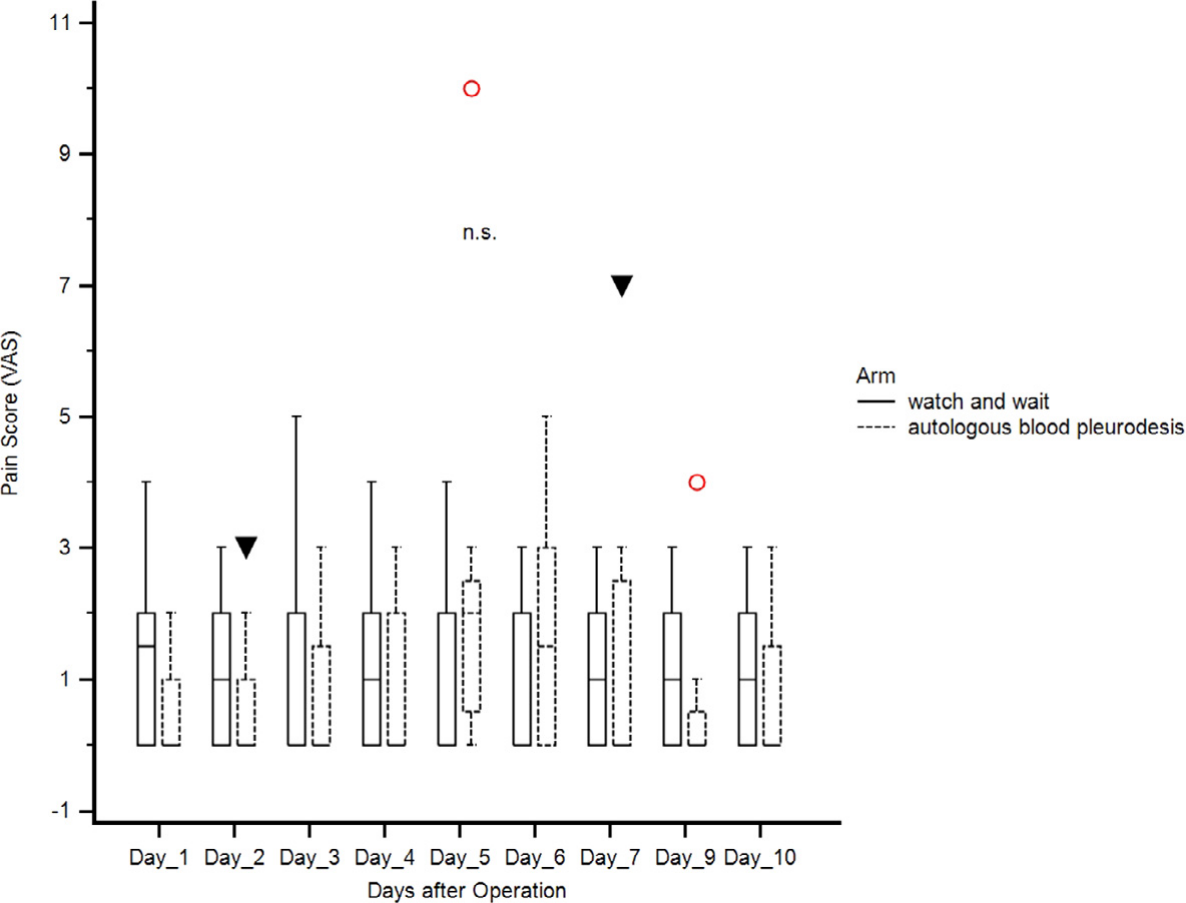

Fig. 1 (Fig. 1) shows pain scores (VAS, visual analog scale) from the first postoperative day until the tenth day after the operation. On day five randomization was performed. Pain levels between both groups did not differ. Pain levels were evaluated with both arms in resting position (watch and wait vs. autologous blood patch pleurodesis, not significant).

**Fig. 2:**
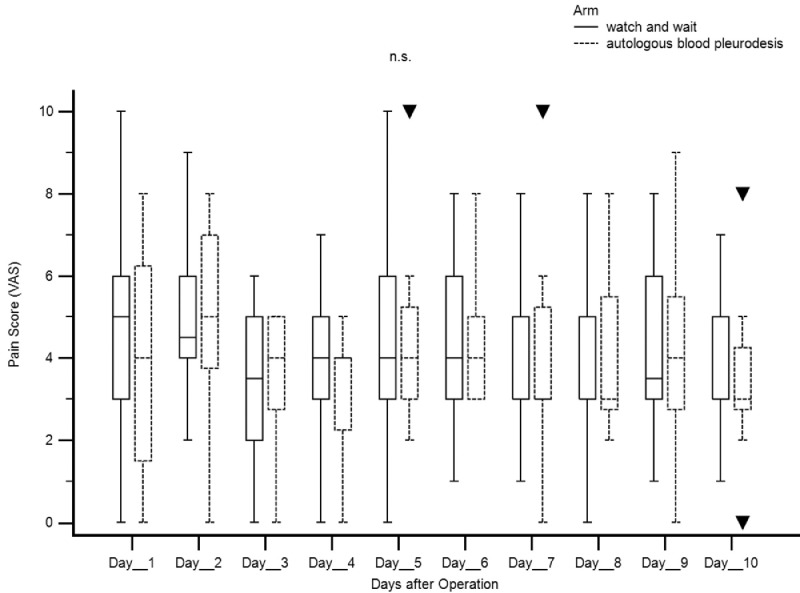

Fig. 2 (Fig. 2) shows pain scores (VAS, visual analog scale) from the first postoperative day until the tenth day after the operation. On day five randomization was performed. Pain levels between both groups did not differ. Pain levels were evaluated while moving both arms (watch and wait vs. autologous blood patch pleurodesis, not significant).

At the first day after operation median pain score in resting position was 1 (range 0 to 4) in the watch and wait arm. On same day median pain score was 0 (range 0 to 3) if patients received autologous blood pleurodesis. From day two until the 10th day after the operation the median pain score was between 4 (range 0 to 10) and 3 (range 0 to 10) in both arms. (Table 2)

**Table 2:** .

	Arm															

	Watch and wait								Autologous blood pleurodesis							
	N	Minimum	Maximum	Mean	Median	SD	RSD	25 - 75 P	N	Minimum	Maximum	Mean	Median	SD	RSD	25 - 75 P

Day 1	14	0,000	4000	1357	1500	1,1507	0,8479	0,000 to 2000	10	0,000	3000	0,778	0,000	1,0929	1,4052	0,000 to 1250
Day 2	14	2000	9000	4786	4500	2,1547	0,4502	4000 to 6000	10	0,000	8000	4889	5000	2,4721	0,5056	3750 to 7000
Day 3	14	0,000	6000	3286	3500	2,0164	0,6137	2000 to 5000	10	0,000	5000	3667	4000	1,7321	0,4724	2750 to 5000
Day 4	14	0,000	7000	3857	4000	1,9556	0,5070	3000 to 5000	10	0,000	5000	3000	4000	1,8028	0,6009	2250 to 4000
Day 5 (Randomization)	14	0,000	10,000	4357	4000	2,7346	0,6276	3000 to 6000	10	2000	10,000	4556	4000	2,3511	0,5161	3000 to 5250
Day 6	14	1000	8000	4286	4000	1,9779	0,4615	3000 to 6000	10	3000	8000	4333	4000	1,6583	0,3827	3000 to 5000
Day 7	14	1000	8000	3857	3000	1,7913	0,4644	3000 to 5000	10	0,000	10,000	4111	3000	2,7588	0,6711	3000 to 5250
Day 8	14	0,000	8000	3571	3000	2,1381	0,5987	3000 to 5000	10	2000	8000	4222	3000	2,1667	0,5132	2750 to 5500
Day 9	14	1000	8000	4071	3500	2,2348	0,5489	3000 to 6000	10	0,000	9000	4222	4000	2,6822	0,6353	2750 to 5500
Day 10	14	1000	7000	3643	3000	1,7368	0,4768	3000 to 5000	10	0,000	8000	3444	3000	2,1858	0,6346	2750 to 4250

Median pain score on first day after the operation when moving was 5 (range 0 to 10) in the watch and wait group and 4 (range from 0 to 8) in the blood pleurodesis group. Between the second and the 10th day after operation median pain score when moving was between 0 (range 0 to 5) and 2 (range 0 to 10). Table 3.

**Table 3:** .

	Arm															

	Watch and wait								Autologous blood pleurodesis							
	N	Minimum	Maximum	Mean	Median	SD	RSD	25 - 75 P	N	Minimum	Maximum	Mean	Median	SD	RSD	25 - 75 P

Day 1	14	0,000	10,000	4571	5000	2,6520	0,5801	3000 to 6000	10	0,000	8000	3889	4000	3,0185	0,7762	1500 to 6250
Day 2	14	0,000	3000	1143	1000	1,0271	0,8987	0,000 to 2000	10	0,000	3000	0,889	0,000	1,3642	1,5348	0,000 to 2250
Day 3	14	0,000	5000	1000	0,000	1,5191	1,5191	0,000 to 2000	10	0,000	3000	0,778	0,000	1,0929	1,4052	0,000 to 1250
Day 4	14	0,000	4000	1286	1000	1,4373	1,1179	0,000 to 2000	10	0,000	3000	0,778	0,000	1,2019	1,5452	0,000 to 2000
Day 5 (Randomization)	14	0,000	4000	1071	0,000	1,4392	1,3433	0,000 to 2000	10	0,000	10,000	2556	2000	3,0046	1,1757	0,750 to 3000
Day 6	14	0,000	3000	0,714	0,000	1,0690	1,4967	0,000 to 2000	10	0,000	5000	1778	2000	1,7159	0,9652	0,000 to 3000
Day 7	14	0,000	3000	1071	1000	1,0716	1,0002	0,000 to 2000	10	0,000	7000	1333	0,000	2,3979	1,7984	0,000 to 2250
Day 8	14	0,000	4000	1214	1000	1,1883	0,9786	0,000 to 2000	10	0,000	5000	1333	0,000	1,9365	1,4524	0,000 to 2500
Day 9	14	0,000	3000	1071	1000	1,0716	1,0002	0,000 to 2000	10	0,000	4000	0,556	0,000	1,3333	2,4000	0,000 to 0,250
Day 10	14	0,000	3000	1071	1000	1,1411	1,0651	0,000 to 2000	10	0,000	3000	0,750	0,000	1,1650	1,5533	0,000 to 1500

## Experimental design, materials, and methods

Study design: Investigator initiated prospective randomized single-center study. Due to the type of intervention blinding was not performed. All patients who demonstrated a persistent air leakage at postoperative day 5 after pulmonary resection were screened for eligibility and asked for consent. Patients were then either randomized to receiving 100 ml autologous blood injected at postoperative days five and six (group A) or to watchful waiting (group B). In case of an expanded lung routinely no suction was applied, however the decision to apply suction in case of insufficient lung expansion was left to the decision of the consultant in charge. The drains were removed as soon as no air leakage was determined for 24 h and fluid production was < 350 ml/24 h.

Routine management: At the end of surgery water seal test was performed to exclude air leakage. In case of observed air leakage all necessary measures were taken to close the fistula. Routinely Ch24 chest tubes were placed. The decision to place one or two chest tubes was depending on the intraoperative situation and the degree of adhesions as well as bleeding tendency. In standard anatomical lung resections usually one posteroapical drainage was used. Digital and conventional chest drainages were used depending on availability and surgeon preference. In standard lung resections – 20 cm H2O suction was applied. In patients with severely emphysematous lungs and after lung volume reduction surgery no suction was used. The decision for drainage removal was based on absence of air leakage for 24 h and fluid production <350 ml/24 h.

Blood patch pleurodesis: Blood patch with autologous blood was performed on ward in aseptic conditions and without any additional analgesia. A quantity of 100 ml blood was taken from a peripheral vein of the upper extremity in sterile conditions and injected immediately in the apical chest tube. No Heparin was added. If possible the chest tube was clamped for a period of 20 min otherwise the chest drain was elevated above the level of the patient´s chest, in order to keep the injected blood within the chest but allow air drainage.

The indication for surgical revision given if a collapsed lung in the chest X-ray despite suction combined with persistent air leakage was observed.

Pain assessment was conducted every day via visual analog scale. We assessed status of pain from the first postoperative day until the tenth day after operation.

Statistical analysis was conducted by using MedCalc software Version 11.6.1.0 (MedCalc software, Broekstraat 52, 9030 Mariakerke, Belgium). Pain score (VAS) was analysed by multivariat analysis (ANOVA). A Bonferroni corrected p value <0.05 was considered as significant.

## Declaration of Competing Interest

(1) All third-party financial support for the work this article:
We report no third-party finical support of any author for this work.
(2) All financial relationships with any entity that could be viewed as relevant to data described in this manuscript;
We report no finical relationship for any author.
(3) All sources of revenue with relevance to this work where payments have been made to authors, or their institutions on their behalf, within the 36 months prior to submission;
We declare no payment for any author.
(4) Any other interactions with the sponsors, outside of the submitted work;
We declare no other interaction with the sponsor.
(5) Any relevant patents or copyrights (planned, pending, or issued);
  There is no patent or copyright related to this work.
(6) Any other relationships or affiliations that may be perceived by readers to have influenced, or give the appearance of potentially influencing, what has been written in this article.
The authors declare that they have no known competing financial interests or personal relationships which have, or could be perceived to have, influenced the work reported in this article.
We declare also no competing interest.
